# Key requirements of a video-call system in a critical care department as discovered during the rapid development of a solution to address COVID-19 visitor restrictions

**DOI:** 10.1093/jamiaopen/ooab091

**Published:** 2021-11-17

**Authors:** Irial Conroy, Aoife Murray, Frank Kirrane, Leonie Cullen, Paul Anglim, Derek O’Keeffe

**Affiliations:** 1 Health Innovation Via Engineering Laboratory, Cúram SFI Research Centre for Medical Devices, Lambe Institute for Translational Research, National University of Ireland—Galway, Galway, Ireland; 2 Department of Medical Physics and Clinical Engineering, University Hospital Galway, Galway, Ireland; 3 Critical Care Department, University Hospital Galway, Ireland; 4 Department of Engineering and Science (Adjunct Lecturer), National University of Ireland—Galway, Galway, Ireland; 5 School of Medicine, College of Medicine Nursing and Health Sciences, National University of Ireland—Galway, Galway, Ireland, and; 6 Lero, SFI Centre for Software Research, National University of Ireland—Galway, Galway, Ireland

**Keywords:** telehealth, critical care, telemedicine, COVID-19 pandemic, intensive care unit

## Abstract

The COVID-19 pandemic necessitated stringent visitor restrictions in critical care departments worldwide, creating challenges in keeping family members connected to patients and clinical staff. Previous studies have examined how hospitals addressed this challenge by repurposing existing tele-ICU systems or by using personal smartphones as a workaround and have analyzed clinical and family feedback. This case report addresses the experience of rapidly implementing a video-call system in the critical care department of a tertiary referral hospital that had no prior video-call system in place, detailing the key requirements in that setting. The 24 requirements were identified via interviews and surveys to both clinical and technical professionals. The top requirements identified were sound and video quality, usability for clinical staff, call control by staff, and patient privacy. From tailoring a video-call solution for this setting, we learned that video-endpoint selection is a key design decision. The initial proposal was to use wireless tablets, but the selection of a large wired video-endpoint allowed us to better address the requirements in the critical care setting. This was based on several characteristics of the large wired video-endpoint, including: high-fidelity video and sound, with directional noise-cancelling; large touch-screen setup for minimal-click navigation; wired as well as wireless connectivity.

## INTRODUCTION

Since the declaration of a pandemic on March 11, 2020, the coronavirus disease (COVID-19) created many challenges for health care systems worldwide.^1^ Due to the high transmissibility of the severe acute respiratory syndrome coronavirus 2 (SARS-CoV-2), many healthcare settings restricted visitors including family members of patients in critical care.^2^ Globally, healthcare providers, particularly critical care professionals, called for solutions to connect family members to critically ill patients and staff, when family could not be physically present at the bedside.^3^^,^^4^ These restrictions raised particular concern as it is widely understood that family play an important role in critical care patient’s recovery.^5^^,^^6^

Telehealth solutions were rapidly adopted across healthcare settings to address many challenges that COVID-19 created.^7–11^ Prior to the pandemic some critical care departments had tele-ICU systems already in place, some utilizing video-call trolleys/carts, but typically used for physiological monitoring and remote specialist input rather than family communications.^11–13^ Many had no video-call systems in place. Previous studies have examined the repurposing of existing tele-ICU systems.^10^^,^^14^ Other studies document solutions based on tablets,^6^^,^^15^^,^^16^ or refer to personal smartphones being used as a workaround.^3^^,^^15^^,^^16^ A few studies have analyzed family and clinician feedback.^17^^,^^18^ In contrast, this case report specifically addresses the experience of implementing a video-call system in a critical care setting with no prior video-call system in place, identifying the key requirements in that setting and how those requirements can be addressed, with particular emphasis on video endpoint selection. This is based on the rapid implementation of a video-call solution, to connect staff, patients, and families, for the critical care department at University Hospital Galway (UHG), Ireland.

## MATERIAL AND METHODS

### Setting

University Hospital Galway (UHG) is a tertiary referral hospital in Galway, Ireland. The critical care department accepted complex patients with COVID-19 from the West and Northwest of Ireland, which have a combined population of ∼750 000. The department has 27 beds, in a mix of “open bays” (three walls and a curtain) and isolation rooms. Prior to March 2020, the critical care department had no formal video-conferencing system used for the purposes of “tele-ICU” or for facilitated video calls with patients’ families.

### Team

An interdisciplinary team was quickly assembled, spanning clinical, academic, and technical-industry partners. The 7-person core team included critical care expertise (1*Nursing; 1*Clinical Engineering), partnering with medical innovation researchers (1*Clinical; 1*Technical/Project Management), and 3 team members from Technology partners (1*Video-conferencing/Networking; 1*Computer programming; 1*Technical Writing/Quality Assurance). The small core team had sufficient expertise to proceed rapidly, with minimal communications overhead, but with wider support to enlist additional expertise as required.

### Iterative development

Similar to other telehealth projects that were implemented at the onset of the pandemic, a rapid, iterative, and highly collaborative approach was taken.^19–22^ The team gained an initial understanding of the “unmet need,” including the requirements specific to the critical care setting, from a meeting with clinical staff and 1:1 interviews with stakeholders. An initial prototype video-call system was customized for the critical care department. This was installed within a few days in the department, where three members of the core team collaborated with hospital staff on testing this and subsequent iterations. The other team-members collaborated remotely on tailoring solution software and configuration.

Initially two different types of video-endpoint were considered—a wireless tablet and a large wired video-endpoint. Testing of both was conducted onsite, and one was chosen based on staff feedback.

### Rollout and support

Hands-on training was provided for existing staff, and training videos were provided for onboarding of additional staff. During the rollout phase, pertinent network and video-call performance metrics were proactively monitored.

### Identifying the key requirements

The key requirements of a video-call system suitable for a critical care setting were identified, considering the requirements identified prior to and during iterative development, but also postrollout feedback related to those requirements. The data were collected from clinical staff via early-stage nonstructured staff interviews, a postrollout clinical survey, and a semistructured interview with a focus group of nurses. The data were collected from technical members of the solution team via a postrollout technical questionnaire.

The data were synthesized and coded by the lead author, using NVivo,^23^ generating a list of requirements. This list of requirements was ranked using scoring by the technical members of the solution team and the lead author. Each requirement was scored on: (1) critical care specificity (2) number of coding references (3) relative importance of addressing; with critical care specificity weighted most heavily. (1, 3) scored by technical members of solution team; (2) scored by lead author.

## RESULTS

### Solution overview

#### High-level requirements

**Figure 1.**
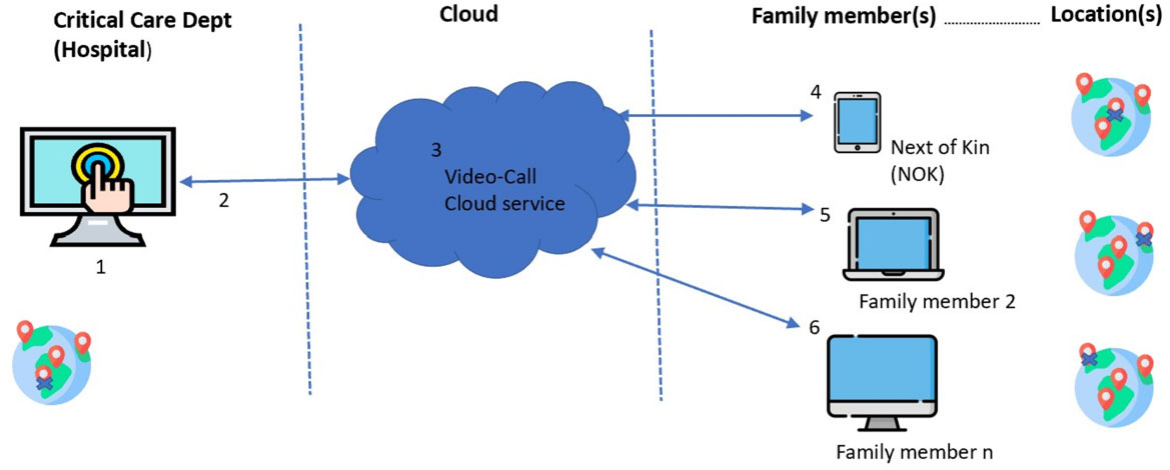
High level system architecture. (1) Video-call endpoint in hospital. (2) Hospital network. (3) Video-call cloud service. (4) Device A, family member 1 (NOK). (5) Device B, family member 2. (6) Device c, family member *n.*

The solution needed to facilitate secure video calls from the patient’s bedside to one or more close family members who may be in separate households. Most importantly for critical care, clinical staff needed the ability to arrange and initiate *ad hoc* virtual visits 24/7 without reliance on administrative staff (Figure  1).

#### Scheduling & workflow

**Figure 2.**
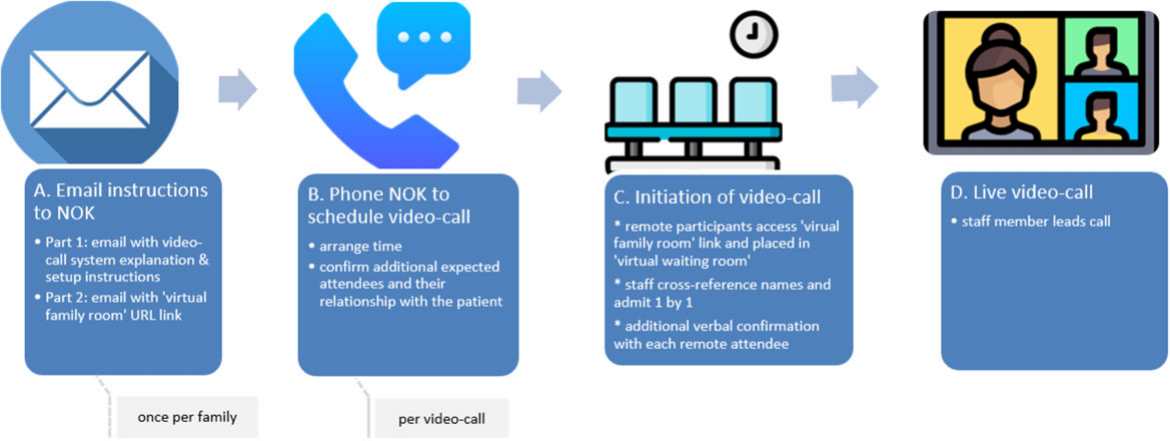
Scheduling and workflow overview.

Scheduling of video-calls was designed to align with existing workflows to minimize burden on staff. Clinical staff arranged video-calls with the designated next of kin (NOK), who was responsible for disseminating to other family members (Figure  2).

#### Critical care video-endpoint

**Figure 3.**
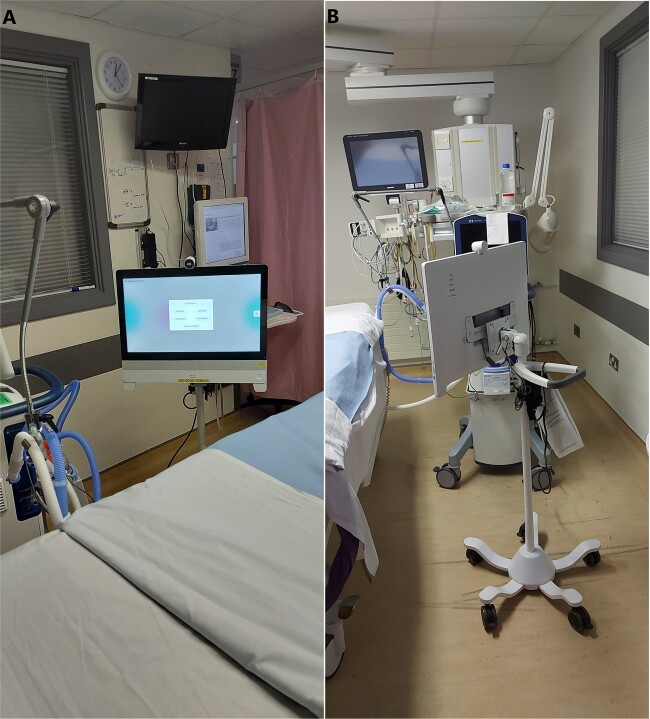
Video-endpoint on stand. (A) Front (B) Rear (and stand).

A 23″ touch-screen video-endpoint that has wired (Ethernet) and wireless (Wi-Fi) connectivity, and high-fidelity audio and visual. It was customized with a menu of “virtual family rooms”, mirroring the existing departmental naming scheme for patient areas or patient rooms. It was also customized for low-touch video-call setup, requiring a minimal number of clicks. It has large “soft”-buttons for the main tasks, the camera has a physical lid and there is a physical mute button. It was mounted on a metal stand creating a freestanding, mobile unit (Figure  3).

#### Video-call cloud service

A commercial cloud video-conferencing platform was used, setup to provide “virtual family rooms”. It was configured to maximize video-call security and patient privacy, including disabling recording, sharing, and chat.

#### Client test devices

Additional devices (9.7″ tablet devices) were provided to facilitate staff training and were used to simulate family-members joining a call. These were configured with a minimal set of applications and managed via a Mobile Device Management (MDM) tool.

#### Technical specifics

The technical details of the solution are available via whitepapers, including video-endpoint and video-call cloud service configuration and customization.^24^ A Data Privacy Impact Assessment (DPIA) was carried out in line with hospital and national General Data Protection Regulation (GDPR) policies, partnering with key hospital stakeholders.

#### Timeline

The first call to a patient’s family took place <2 weeks after project inception, and the full system rollout was completed in <3 weeks.

### Key requirements

The postrollout clinical survey had 17 respondents (*n* = 17), the focus group consisted of four nurses, and the postrollout technical questionnaire was completed by all four technical members of the solution team.

24 requirements of a video-call system suitable for a critical care setting were identified. The top 12 are described in Table  1, in descending rank order. The full list is in Supplementary MaterialTable S1.

**Table 1.** Key requirements of a video-call system in critical care setting

Category	Description
Sound quality	Ensuring effective audio communications, considering: background noise (respiratory ventilators, bedside medical equipment, nearby beds); staff may be wearing PPE.
Video quality	Ensuring effective visual communications, considering: need bed bound patient to easily see multiple family members; video-feed quality issues would cause additional stress for remote family; nonverbal’s particularly important to convey.
Usability (for staff)	Ensuring the system is easy-to-use for staff, considering: staff need unimpeded mobility around the bedside and handsfree operation during video-call; staff may be wearing gloves
Patient privacy	Ability to manage patient privacy: staff control of family member access; removal of recording options; adherence to Data Privacy regulations eg, General Data Protection Regulation (GDPR).
Staff resourcing/workload	Ensuring system adds minimal additional workload for staff.
Infection control	Ability to clean/disinfect the device(s) to adhere to Infection Control guidelines.
Call control (by staff)	Ability for staff to easily control the call (including call initiation/termination, camera/audio).
Reliability	Ensuring system is stable and reliable (especially considering the importance of some of the calls eg, End of Life scenarios).
Staff training	Ensuring system has low training requirements.
	Ensuring system provides the ability for staff to easily simulate the end-to-end scenario.
Existing critical care processes	Ensuring easy alignment/integration with existing local critical care protocols.
	Agreeing ownership for new activities (eg, family notification).
Physical access (solution team)	Ability for the solution team to implement infrastructure changes (eg, device installation, networking changes) that require physical access to the critical care department.
Network	Ensuring sufficient network access (including Ethernet access) and bandwidth for video-calls.

Descending rank order.

#### Sound quality

The system needed to accurately capture speech from staff and a bed bound patient, whilst minimizing the impact of background noise—from respiratory ventilators, bedside medical equipment, as well as conversations from nearby beds. Staff were wearing masks, respirators, and face shields—this Personal Protective Equipment (PPE) made audio communication more difficult.

#### Video quality

The system needed to allow a bed bound patient to easily see multiple family members at the same time. The system needed to provide a high-quality video feed for remote family members to see the hospital environment and the patient, and minimize the possibility of causing additional stress due to video quality issues. Some patients had limited ability to communicate, so it was particularly important to adequately convey facial expressions and other nonverbal communications.

#### Usability for clinical staff

Staff needed the ability to move around the bedside and to conduct the video-call handsfree—to allow them to assist the patient or carry out other tasks, as necessary. The use of gloves made it harder to operate electronic devices. Staffs were under significant pressure preparing for a surge in COVID-19 cases, particularly with upskilling on new equipment and training of redeployed staff. Hence ease of use was even more important to ensure system adoption.

#### Call control by staff and patient privacy

Communication with patients’ families is always a key part of critical care and often includes sensitive discussions. Staff needed confidence that they could easily and reliably control the basics of the video-call for these communications, particularly starting and stopping the call and quickly turning the camera and audio on and off. Staff needed to be able to securely control the identification of family members, and their admission to the call at an appropriate time to ensure patient privacy.

In addition to gathering data on the requirements, the interviews and surveys also gathered anecdotal feedback from staff on their overall experience of communicating with families of critical care patients while stringent visiting restrictions were in place. Prior to the introduction of the video-call system one staff nurse commented, *“Specifically for ICU* [Intensive Care Unit]*, our patients can be sedated and ventilated, and I think families can experience a lack of control, as they have to trust people they have never met to care for their loved one*”. After using the video-call solution for a few weeks, a staff nurse noted, “*to physically see the person—it tells them* [the family] *so much more about how they* [the patient] *are than what we’ll say. Literally, they trust us a little better, they can back up what we’re telling them with physically seeing them themselves*”.

## DISCUSSION

The system has been successfully deployed and has been used frequently by staff during the pandemic and the department anticipate using it after the lifting of visiting restrictions, particularly for families abroad or at a distance. Careful selection of video-endpoints and adherence to local policies and regulations are particularly important in the critical care environment.

### Video-endpoint

The video-call solution comprised multiple components and each contributed toward addressing the requirements—the top 12 requirements were addressed by a combination of these solution components: video-endpoint, network, workflow integration, IT integration, documentation, and training. A mapping of these solution components to requirements is provided in Supplementary MaterialTable S2. This mapping identifies the video-endpoint as the main contributor toward addressing the requirements.

For the video-endpoint, we chose a large wired video-endpoint in preference to a wireless tablet, based on staff feedback. A tablet-based solution would have provided a workable solution, but selection of the large wired video-endpoint enabled the solution to address more of the requirements, based on several device characteristics.

#### Sound quality

The large wired video-endpoint provides a directional microphone and noise-cancelling. This allowed it to sufficiently filter the background noise, to allow staff (despite PPE) or patients with weaker voices (or poor voice projection) to effectively communicate to remote family.

#### Video quality

The chosen video-endpoint has a 23″ high-definition (HD) screen and high-fidelity camera. Staff noted that this provided high-quality video, for various positioning of the freestanding unit (eg, at end of the bed), including when rooms/bays were brightly lit.

#### Usability, call control

The chosen endpoint was customized to be freestanding and low-touch—these were essential characteristics in the critical care department. Most critical care patients are not able to independently hold tablet devices, and a freestanding device allows staff to independently move/leave, and could enable less rushed video calls. A freestanding device also gives the ability to setup calls to allow the family to watch the patient sleeping.

#### Reliability, network

The chosen video-endpoint provides the option of connecting to a network via wired (Ethernet) and/or wireless (Wi-Fi). Our solution utilized both options, setting up a wired connection as the default and a wireless connection as a fallback. From a technical perspective, this provided a reliable network connection on the hospital side of the video-call. Interestingly, some staff commented that they felt a greater sense of confidence initiating sensitive calls (eg, End of Life scenarios) with a wired connection in place.

### Adherence to legal requirements and hospital policies

Prior to the pandemic, video-conferencing systems specifically for family communication were not commonly used. When introducing similar systems, hospitals and staff have a responsibility to ensure patient privacy and to adhere to data protection policies as well as accepted legal and ethical guidance surrounding the appropriate use of video-conferencing in the healthcare setting. These requirements will differ depending on the physical environment of the unit (eg, open floor plans), local policies, and regional regulations.^25^ We found that partnering closely with key hospital stakeholders, with prior experience of pertinent regulations, expedited the process (eg, GDPR/DPIA).

### Limitations

While the system meets the needs of patients, staff, and families, there are limitations and scope for better iterations. The wired network connection requires physical wiring & software configuration and restricts the mobility of the video-endpoints. For this project, we deemed that the benefits, particularly in terms of connection reliability and staff confidence, outweighed those limitations.

Solution workflow could be streamlined by integration into the Electronic Health Record (EHR). The solution does not provide an automated process for handling when patients move area/bed.

Staff highlighted that the solution does not replace the tactile interaction between family and patient, and the reassurance that it provides.

The project was delivered by volunteers, with donated equipment. In a broader rollout cost-effectiveness may be a limitation, given the use of high-end video-call endpoints. This is beyond the scope of this report.

The main limitation of this case report is that it is based on implementing a video-call system in the critical care department of just one hospital. The report is also limited in the following aspects: just two video-endpoint types were evaluated as part of the project; ranking of requirements is primarily based on scoring by a small team (technical members of solution team, *n* = 4); descriptive statistics of system usage are not supplied; solution options from multiple vendors were not evaluated, as the project was delivered by a coalition of volunteers.

### Further research

Further research is warranted in this area: studies across multiple sites, over longer timeframes, with direct feedback from family and patients, comparing more video-endpoint types.

More broadly, recent studies around telepresence and telepresence robots have shown their potential in clinical settings.^26^^,^^27^ Advances in those areas allied with advances in the field of haptics,^28^^,^^29^ may help to address the unmet need around tactile interaction between patients and families, identified above—this warrants further research.

## CONCLUSION

Video-call solutions have been rapidly adopted across healthcare settings to address many challenges that COVID-19 created. Whereas at the start of the pandemic generic video-call solutions were acceptable and have proven invaluable, one would expect subsequent solution generations to be tailored for different healthcare settings. From tailoring a video-call solution for a critical care setting, we learned that video-endpoint selection is a key design decision. The initial proposal was to use wireless tablets, but the selection of a large wired video-endpoint allowed us to better tailor the solution to the critical care setting and better address the requirements in that specific environment to meet the needs of patients, staff, and families.

## FUNDING

This publication has emanated from research supported in part by a grant from Science Foundation Ireland (SFI) and the European Regional Development Fund (ERDF) under grant number 13/RC/2073_P2 (AM, DOK) and 13/RC/2094_P2 (DOK).

## AUTHOR CONTRIBUTIONS

All authors meet the four criteria of authorship in accordance with ICMJE guidelines. IC, AM, PA, and FK contributed to conception or design of the work. IC, FK, and LC contributed to data acquisition. IC, AM, PA, DOK, and FK contributed to data analysis and interpretation. IC contributed to literature search. IC and AM contributed to drafting the article. All authors contributed to critical revisions, approved the final version to be published, and agreed to be accountable for all aspects of the work.

## SUPPLEMENTARY MATERIAL

Supplementary material is available at *JAMIA Open* online.

## Supplementary Material

ooab091_Supplementary_Data
