# Supplementary material for: Key requirements of a video-call system in a critical care department as discovered during the rapid development of a solution to address COVID-19 visitor restrictions
Source: JAMIA Open. 2021 Nov 17;4(4):ooab091. doi: 10.1093/jamiaopen/ooab091 (PMC8599714; doi:10.1093/jamiaopen/ooab091)
Supplement: ooab091_Supplementary_Data [file ooab091_supplementary_data.zip › SupplementaryMaterial_Table2.docx]

**Table 2**: Video-call solution characteristics that address the requirements in a critical care setting

| Requirement | Solution Component | Solution Characteristics |
| --- | --- | --- |
| Sound Quality | Video-endpoint | (i) High-fidelity audio (speaker and microphone).  (ii) Directional microphone and noise-cancelling: filter background noise. |
| Video Quality | Video-endpoint | (i) Large screen (23”)  (ii) High-definition (HD) screen  (iii) High-fidelity camera  (iv) Freestanding, mobile unit  Combination of (i,ii,iv) allows bed bound patient to view multiple family members easily |
| Usability (for staff) | Video-endpoint | (i) Large touchscreen.  (ii) Low-touch: minimal number of button presses to conduct a video call.  (iii) Freestanding, mobile unit.  (iv) Large buttons for the main tasks.  (v) Physical labels on camera and key buttons (e.g. mute).  Combination of (i-iii) enable staff to move around the bed/attend to the patient |
|  | Workflow integration | (i) Naming scheme (used for menu of ‘Virtual Family Rooms’ on video-call endpoint and as part of ‘Virtual Family Room’ URL) aligns with existing departmental naming scheme for patient areas/bedspaces. |
| Patient Privacy | Video-endpoint | (i) Family members in 'Virtual Waiting Room' until staff member allows them in (1-by-1). (ii) Recording disabled. |
|  | Documentation | (i) Email templates: Disclaimers in emails that only close family members allowed, and that recording not permitted. |
| Staff Resourcing/Workload | Workflow integration | (i) Clinical staff can arrange and initiate ad hoc virtual visits 24/7 without administration staff (based on supplied email templates).  (ii) Minimal training required. |
| Infection Control | Video-endpoint | (i) Video-endpoint can be cleaned/disinfected to hospital guidelines. |
| Call Control (by staff) | Video-endpoint | (i)'Soft'-buttons for main tasks (start/stop).  (ii) Custom menu of 'Virtual Family Room's.  (iii) Camera with physical lid.  (iv) Physical mute button.  (v) Physical labels on camera and key buttons (e.g. mute). |
| Reliability | Network | (i) Ethernet setup as primary network access mechanism, with Wi-Fi access setup as fallback. |
| Staff Training | Training | (i) Usage instructions, documentation & training video supplied.  (ii) Client test devices enable staff to simulate end-to-end video-calls (from both sides: hospital & family) |
| Existing Critical Care Processes | IT integration | (i) Admissions process modified to gather Next of Kin (NOK) email. (ii) Emails to family added as templates in existing email system. |
| Physical Access (Solution Team) | Other | (i) Delivered 'alpha' version of customised video-endpoint within initial access time-window, with remote access option to allow time-limited remote development |
| Network | Network | (i) Capacity Planning: Assessed video-call bandwidth requirements and verified available bandwidth. |
|  | Network, Video-endpoint | (i) Wired: Enabled, configured, and labelled Ethernet ports at agreed locations. (ii) Wireless: Installed additional Wi-Fi access points. |
