# Supplementary material for: Key requirements of a video-call system in a critical care department as discovered during the rapid development of a solution to address COVID-19 visitor restrictions
Source: JAMIA Open. 2021 Nov 17;4(4):ooab091. doi: 10.1093/jamiaopen/ooab091 (PMC8599714; doi:10.1093/jamiaopen/ooab091)
Supplement: ooab091_Supplementary_Data [file ooab091_supplementary_data.zip › SupplementaryMaterial_Table1.docx]

**Table 1**: Requirements of a video-call system in a critical care setting

| Category | Description |
| --- | --- |
| Sound Quality | Ensuring effective audio communications, considering: background noise (respiratory ventilators, bedside medical equipment, nearby beds); staff may be wearing PPE. |
| Video Quality | Ensuring effective visual communications, considering: need bed bound patient to easily see multiple family members; video-feed quality issues would cause additional stress for remote family; non-verbal’s particularly important to convey. |
| Usability (for staff) | Ensuring the system is easy-to-use for staff, considering: staff need unimpeded mobility around the bedside and handsfree operation during video-call; staff may be wearing gloves |
| Patient Privacy | Ability to manage patient privacy: staff control of family member access; removal of recording options; adherence to Data Privacy regulations e.g. General Data Protection Regulation (GDPR). |
| Staff Resourcing/Workload | Ensuring system adds minimal additional workload for staff. |
| Infection Control | Ability to clean/disinfect the device(s) to adhere to Infection Control guidelines. |
| Call Control (by staff) | Ability for staff to easily control the call (including call initiation/termination, camera/audio). |
| Reliability | Ensuring system is stable and reliable (especially considering the importance of some of the calls e.g. End of Life scenarios). |
| Staff Training | Ensuring system has low training requirements.  Ensuring system provides the ability for staff to easily simulate the end-to-end scenario. |
| Existing Critical Care Processes | Ensuring easy alignment/integration with existing local critical care protocols.  Agreeing ownership for new activities (e.g. family notification). |
| Physical Access (Solution Team) | Ability for the solution team to implement infrastructure changes (e.g. device installation, networking changes) that require physical access to the critical care department. |
| Network | Ensuring sufficient network access (including Ethernet access) and bandwidth for video-calls. |
| Legal Concerns (Staff) | Legal concerns raised by staff |
| Technical Proficiency (Family) | Family members have varying levels of technical proficiency. |
| Non-Technical Support (Family) | Ability to support family members on non-technical aspects (e.g. emotional support) |
| Maintainability | Ensuring system is maintainable |
| Technical Proficiency (Staff) | Staff members may have varying levels of technical proficiency. |
| Mobility | Ability to move the system between bedspaces, and within the bedspace area |
| Ethical Concerns (Staff) | Ethical concerns raised by staff |
| Security | Ensuring video-calls are secure |
| Scheduling | Ability to schedule video-calls ad hoc as well as for specific times |
| Internet Access (Family) | Some family members may have poor/no Internet Access |
| Email Access (Staff) | Staff may have limited access to email |
| Technical Support (Family) | Facility to support technical queries from families |

descending rank order
